# *Hsmar1* Transposition Is Sensitive to the Topology of the Transposon Donor and the Target

**DOI:** 10.1371/journal.pone.0053690

**Published:** 2013-01-14

**Authors:** Corentin Claeys Bouuaert, Ronald Chalmers

**Affiliations:** School of Biomedical Sciences, University of Nottingham, Nottingham, United Kingdom; Institute of Enzymology of the Hungarian Academy of Science, Hungary

## Abstract

*Hsmar1* is a member of the *Tc1-mariner* superfamily of DNA transposons. These elements mobilize within the genome of their host by a cut-and-paste mechanism. We have exploited the *in vitro* reaction provided by *Hsmar1* to investigate the effect of DNA supercoiling on transposon integration. We found that the topology of both the transposon and the target affect integration. Relaxed transposons have an integration defect that can be partially restored in the presence of elevated levels of negatively supercoiled target DNA. Negatively supercoiled DNA is a better target than nicked or positively supercoiled DNA, suggesting that underwinding of the DNA helix promotes target interactions. Like other *Tc1-mariner* elements, *Hsmar1* integrates into 5′-TA dinucleotides. The direct vicinity of the target TA provides little sequence specificity for target interactions. However, transposition within a plasmid substrate was not random and some TA dinucleotides were targeted preferentially. The distribution of intramolecular target sites was not affected by DNA topology.

## Introduction

DNA transposons are specialized genetic entities that mobilize and amplify within a host genome. Their success as genomic parasites is highlighted by the abundance and ubiquity of transposase genes in nature [1]. The impact that transposons have had on the evolution of genomes is such that they are considered as one of the major forces driving evolution [2], [3].

DNA transposons are useful tools to manipulate genomes. They have been used in a variety of biotechnological applications including transgenesis and insertional mutagenesis and offer promises for gene therapy [4]. The *Tc1-mariner* transposons are amongst the most extensively exploited elements because they involve simple and versatile components and they are active in a wide range of hosts, including vertebrates [5], [6].

**Figure 1:**
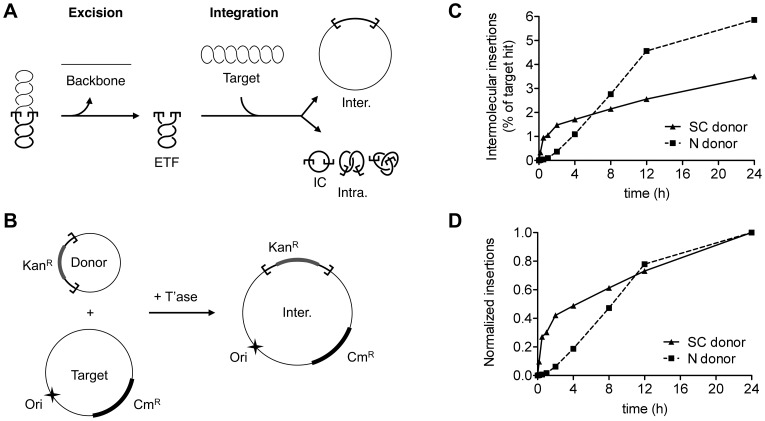
The topology of the transposon donor affects integration. **A**, The *Hsmar1* transposition reaction. Double-strand cleavage at both transposon ends liberates the plasmid backbone, which is an end product of the reaction, and the excised transposon fragment (ETF), which goes on to integrate into a target. Intermolecular insertions (Inter.) may target any DNA present in the reaction, like a target plasmid provided. Intramolecular insertions (Intra.), within the transposon itself, produce a series of topologically complex transposon circles [7]. IC, unknotted inversion circle. **B**, The *in vitro* ‘hop’ assay for the quantification of intermolecular integration events. The transposon donor (pRC704) encodes a kanamycin resistance marker (Kan^R^) flanked by *Hsmar1* transposon ends. *In vitro* transposition is performed in the presence of a target plasmid (pACYC184) encoding a chloramphenicol resistance marker (Cm^R^). After transformation in *E. coli*, the intermolecular transposition efficiency is deduced by dividing the number of colonies obtained after selection on Kan+Cm by the number of colonies obtained on Cm alone. The donor plasmid has a conditional origin of replication that does not function in the recipient strain. This serves to eliminate any bias introduced by double transformation events in which a cell receives copies of both the donor and the target plasmid. The target used is a dimeric form of the pACYC184 plasmid, which allows recovering integration events that are not biased by the disruption of essential regions within the target. T’ase, transposase. **C**, **D**, A time course analysis was performed using a supercoiled (SC) and nicked (N) transposon donor in the presence of a supercoiled target. Intermolecular integration events were recovered by a genetic ‘hop’ assay (see Materials and Methods). **C**, The percentage of target hit is plotted against time. Because transposon and target are in equimolar concentrations, the percentage of target plasmids that are hit by a transposon also corresponds to the percentage of transposons that perform intermolecular integration into the target. **D**, Insertions are normalized to their maximal value.

*Tc1-mariner* transposons mobilize via a cut-and-paste mechanism (Figure 1A). Transposition is preceded by the assembly of the paired-ends complex (PEC), where the two ends of the transposon are brought together into a higher-order complex also called the transpososome [7]. Upon PEC formation the transposase catalyses the formation of a double-strand break at both transposon ends [8]. This excises the transposon from the donor sequence. The 3′-ends of the transposon are subsequently transferred 5′ of a TA target site, generating single-strand gaps flanking the newly inserted transposon [5], [9]. These gaps are repaired by host-encoded machinery and lead to the duplication of the TA target dinucleotide at either side of the element.

Except for the stringent requirement for a TA dinucleotide, target site selection in *Tc1-mariners* is considered essentially random [10], [11], [12], [13], [14]. The immediate vicinity of the TA dinucleotide usually provides little specificity. Nevertheless, biased target selection has been observed for several *Tc1-mariner* elements, both *in vivo* and *in vitro* [10], [13], [15], [16], [17]. Correlations between preferred target sites and predicted physical properties of the local DNA revealed that DNA bendability is likely a factor favoring target interactions of the *Sleeping Beauty* and *Himar1* transposons [11], [13], [16], [18]. In contrast, *Mos1* integration into a plasmid carrying the Tn*9* chloramphenicol acetyltransferase (*cat*) gene hot spot did not correlate with DNA bendability but required supercoiling [17].

The functional state of the target may also affect transposon integration. For example, some transposons display a tendency to integrate into active genes. This is the case of the P-element that preferably integrates near transcriptional start sites [19]. Other transposons like *Mu* or Tn*10* avoid integrating into transcriptionally active regions, which helps reducing their disruptive effects to the host [20]. The specificity of *Tc1-mariner* integration has not been thoroughly characterized on a genomic scale, but studies with *Sleeping Beauty* and *Hsmar1* did not reveal an integration bias for active genes [11], [12]. Nucleosomes also influence the insertion specificity of transposons. Some transposons integrate preferentially into nucleosome-free regions, like *Hermes* and the *Tf1* retrotransposon, while others specifically target nucleosome-occupied sequences, like the *Ty1* retrotransposon [21], [22], [23].

Another source of integration bias commonly observed with chromosomal copies of transposons is their tendency to integrate target sites located in close proximity of their original position. This phenomenon, referred to as ‘local hopping’, most likely reflects the elevated probability for a transposon to interact with target DNA located nearby the excision site due to rapid interactions with the target after excision and slow diffusion of the excised transposon due to macromolecular crowding. The *Sleeping Beauty* and the *hAT* elements *Tol2* and *Hermes* are prone to local hopping, whereas *PiggyBac* appears to be less so [11], [24], [25], [26].

*Hsmar1* was active in the human genome from about 50 to 37 million years ago [27]. During that time about 200 copies of full-length element were generated together with thousands of copies of a non-autonomous miniature derivative [28]. In addition, one copy of the transposase gene was domesticated by the human genome and evolved under purifying selection as part of the SETMAR gene [29]. SETMAR is involved in DNA repair but its precise function remains unclear [30]. Nevertheless, several activities have been reported including activities derived from the ancestral *Hsmar1* transposase [31]. Here, we are working with a resurrected copy of the *Hsmar1* transposase, which was deduced by computational analysis of the fossil *Hsmar1* elements [12]. This resurrected *Hsmar1* transposase is highly active *in vitro* [32]. Taking advantage of this system, we have previously shown that DNA supercoiling accelerates *Hsmar1* transposition by bringing the transposon ends in a favorable configuration for synapsis [7]. This provides the transposase with a ‘topological filter’ that allows the transposase to selectively synapse transposon ends that are in the appropriate inverted repeat configuration. We proposed that this topological filter serves to limit transposon-induced genome instability and to couple transposition to cellular events.

Here, we have investigated the effect of DNA supercoiling on the integration step of *Hsmar1* transposition. We show that the topology of both the transposon donor and the target affect integration. Negatively supercoiled DNA is the preferred target for insertion and helps drive the otherwise slow integration of a relaxed transposon. Like other *Tc1-mariner* elements, *Hsmar1* shows little sequence specificity in the vicinity of the target TA. Nevertheless, some target sites are preferred. Supercoiled and nicked transposon donors have similar biases for intramolecular integration sites suggesting that the distribution of integration events is unaffected by the topology of the target.

## Materials and Methods

### Media and Bacterial Strains

Bacteria were grown in Luria-Bertani (LB) media at 37°C. The following antibiotics were used at the indicated concentrations: ampicillin (Amp), 100 µg/ml; kanamycin (Kan), 50 µg/ml; chloramphenicol (Cm), 34 µg/ml. The following Escherichia coli strains were used: RC5024 (identical to DH5α) {endA1 hsdR17 glnV44 thi-1 recA1 gyrA relA1 Δ(lacIZYA-argF)U169 deoR [φ80dlac Δ(lacZ)M15]}, RC5081 (identical to S17.1λ pir), and RC5036 (identical to BL21(DE3)) {F– ompT gal dcm lon hsdSB(rB- mB-) λ(DE3 [lacI lacUV5-T7 gene 1 ind1 sam7 nin5])}.

### Expression Vector and Plasmid Substrates

Plasmid pRC880 is a pMAL-c2x based expression vector that contains the gene encoding the reconstructed ancestral version of the *Hsmar1* transposase [32]. Standard transposition reactions contained the plasmid pRC650 or pRC704. The genetic ‘hop’ assay used pRC704 as transposon donor and a dimeric form of pACYC184 (chloramphenicol and tetracycline) as target. pRC704 encodes a 2.3 kb kanamycin-resistance *Hsmar1* transposon and a 0.8 kb plasmid backbone encoding the R6K conditional origin of replication. When the transposon donor or the target was relaxed the corresponding plasmid pRC650 or pRC704 (donors) or pACYC184 (target) were digested with the nicking endonuclease NbBsrDI (New England Biolabs). NbBsrDI cuts the donor plasmids at several sites located hundreds of base pairs away from the transposon ends. When the target was positively supercoiled, the pACYC184 plasmid was treated with reverse gyrase as described previously [7], [33]. After treatment with reverse gyrase or NbBsrDI, the DNA was phenol-chloroform extracted, ethanol precipitated and resuspended in TE buffer. The DNA was quantified using a Nanodrop spectrophotometer before sample preparation.

### Transposase Purification and Standard Transposition Assay

Protocols for the purification of the *Hsmar1* transposase and the *in vitro* transposition assay have been described previously [32]. Briefly, the *Hsmar1* transposase was expressed as a maltose-binding protein fusion in *E. coli* cells harboring the pRC880 vector and purified by affinity chromatography on amylose resin (New England Biolabs), followed by ion-exchange chromatography on a MonoS HR5/5 column (Amersham Pharmacia). Transposition reactions with the standard substrate pRC650 contained 6.7 nM transposon donor and 20 nM transposase in 20 mM Tris-HCl buffer pH 8, 100 mM NaCl, 10% glycerol, 2 mM DTT and 2.5 mM MgCl_2_. Transposase was diluted in reaction buffer and was always the last component added to the reaction mixture. After incubating at 37°C for up to 24 h, reactions were made 20 mM in EDTA, 0.1% SDS and heated at 75°C for 30 minutes. DNA was recovered by ethanol precipitation and analyzed by agarose gel electrophoresis.

### Genetic ‘hop’ Assay

The genetic hop assay was also described previously [32]. The reaction conditions were as above, except for the following modifications. Unless stated otherwise, 20 µl reactions contained 43 ng pRC704 transposon donor (1 nM), 112 ng dimeric pACYC184 target (1 nM) and 10–15 nM transposase. Reactions were incubated at 37°C for up to 24 h then stopped and extracted with phenol-chloroform. DNA was recovered by ethanol precipitation and resuspended in TE buffer. One tenth of the reaction was transformed into DH5α competent cells. After transformation, 1/100 of the mixture was spread on LB-chloramphenicol plates. The number of colonies provided a measure of the total amount of target DNA recovered. The remainder of the transformation mixture was spread on LB-chloramphenicol plus kanamycin plates and the number of colonies was used to quantify the proportion of the target plasmids that had received a transposon insertion. The donor plasmid has a conditional origin of replication that does not function in the recipient strain. This serves to eliminate the confounding effect of double transformation events with donor and target. No colonies were obtained if the transposon donor, the target or the transposase were omitted. DNA sequencing analysis of the junction between the transposon donor and the target confirmed that the colonies represent true transposition events. All of the 54 insertions tested were precise and carried a duplicated target TA dinucleotide. Half of the transposition reaction was also analyzed on a 1.1% agarose gel. After electrophoresis the gel was stained with SYBR Green I and recorded on a fluorimager.

## Results

### Donor DNA Topology Affects Integration

We used an *in vitro* ‘hop’ assay to quantify intermolecular transposition of *Hsmar1* from supercoiled (SC) and nicked (N) donor plasmids (Figure 1B). In this assay, the transposon donor (pRC704) encodes a kanamycin resistance marker flanked by *Hsmar1* transposon ends. The reaction is performed in the presence of a target plasmid (dimeric pACYC184) that encodes a chloramphenicol resistance marker. Transposition events, from donor to target, are subsequently recovered by genetic transformation in *E. coli*.

Transposition reactions with a supercoiled or a nicked transposon donor were initiated at time zero and aliquots for transformation were withdrawn at the indicated times (Figure 1C and D). Initially, the rate of transposition was higher with a supercoiled donor than with a nicked donor. The reaction had reached 50% completion after 4 h with a supercoiled substrate, but only after 8 h with a nicked substrate. However, after 24 h the total amount of intermolecular events obtained with the nicked donor was almost twice that with the supercoiled donor.

In the *in vitro* assays, almost 100% of the substrate is converted into products [32]. Since the transposon donor and target plasmids are equimolar, the proportion of target plasmids that receive insertions is equivalent to the proportion of the transposon that goes on to perform intermolecular integration in the target. The remainder of transposons, which corresponds to 94 and 97% for the nicked and supercoiled substrates, respectively, integrates into other DNA sequences present. These include unreacted donor plasmids or the transposon DNA itself. The vast majority of integration events are intramolecular [32].

### Increasing Target Concentration Drives Intermolecular Integration

**Figure 2:**
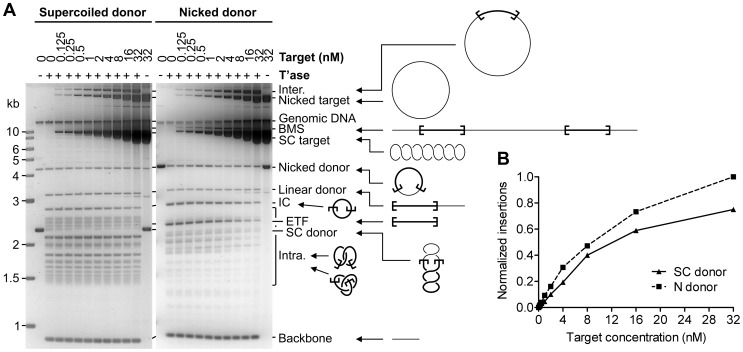
Intermolecular integration events increase with target concentration. **A**, Transposition reactions with increasing amounts of target DNA (dimeric pACYC184 plasmid) were performed using 1 nM supercoiled (SC) or nicked (N) transposon donor (pRC704). Reactions were incubated for 24 h. The products were analyzed by agarose gel electrophoresis. The observed products were previously identified by one- and two-dimensional gel electrophoresis methods [7], [32]. The identity of the product of intermolecular integration of the transposon within the target plasmid (Inter.) was confirmed by cutting the band out of the gel, transforming the DNA in *E. coli* and analyzing plasmid DNA prepared from several clones by restriction digestion and DNA sequencing. The products of intramolecular integration of the transposon (Intra.) form an array of knotted and catenated products that vary in the numbers of supercoiling nodes that were trapped during integration [7], [32]. The intramolecular integration product of lowest electrophoretic mobility is the unknotted inversion circle (IC), which trapped zero supercoiling nodes. The diffuse band running between the IC and the SC donor represents the first catenane, which trapped two supercoiling nodes. Each catenane consists of a pair of gapped circles together equaling the size of the transposon. Although all the products within this diffuse band have the same molecular weight and topological complexity, the gel is able to resolve a pair of circles of similar sizes from a pair of circles of very different sizes. Trapping a third supercoiling node leads to the formation of the first knot, which runs as a discrete band below the SC donor. Knots and catenanes of increasing electrophoretic mobility then alternate together with increased topological complexity. With a nicked donor the predominant intramolecular integration products are the IC and deletion circles. Deletion circles are unlinked pairs of gapped circles together equaling the size of the transposon. The 2.3 kb linear excised transposon fragment (ETF) is not detected with the supercoiled transposon donor because transposon integration with this substrate is rapid and efficient. Nevertheless, the ETF can be detected at early time points in synchronized transposition reactions [7], [32]. BMS is an intermolecular integration product that results from bimolecular synapsis between two transposon donors and forms a linear species of the size of a target plasmid plus two transposon donors. The BMS product is not detected with the supercoiled donor because DNA supercoiling promotes the constrained synapsis of transposon ends in plectonemic supercoiling nodes, reducing the number of bimolecular synaptic events [7]. DNA preparations of the supercoiled donor and target plasmids always contain some amount of nicked products. **B**, Intermolecular insertions of the reactions in panel A were recovered by transformation in *E. coli* and selection on appropriate medium. The number of colonies recovered were normalized to the maximal value and plotted.

The *Hsmar1* reaction, with a supercoiled or a nicked substrate, was titrated with increasing amounts of a supercoiled target plasmid and analyzed by gel electrophoresis (Figure 2A). For these reactions the standard incubation time of 6 to 8 hours was extended to 24 h to allow the slow reaction with the nicked donor to reach completion. Increasing the target DNA concentration over a 500-fold range did not significantly affect the extent of excision, judging from the amount of vector backbone produced in each case. However, the amount of intermolecular transposition increased with increasing concentration of the target. This suggests that the high proportion of intramolecular events detected in the *in vitro* reactions is at least in part due to the low concentration of target DNA in vitro. Presumably, intramolecular target sites are favored because they are covalently attached to the transpososome and are therefore presented at a high relative concentration.

At low target concentrations, the excised transposon fragment (ETF) originating from the nicked donor was detected at elevated levels but disappeared with increasing target concentration (Figure 2A, right panel). In contrast, the ETF was not visible with the supercoiled donor. This shows that a relaxed transposon is less efficient in integration than a supercoiled transposon. This deficiency in integration can be partially restored in the presence of elevated levels of supercoiled target.

The nicked donor also generates an integration product not present in the reactions with supercoiled substrate. It results from bimolecular synapsis (BMS) between transposon ends on two different transposon donor molecules. Cleavage linearizes both donors and the target upon insertion. BMS events are not recovered with supercoiled substrates because DNA supercoiling favors intramolecular synapsis by juxtaposing the transposon ends in the plectosome [7]. In the absence of DNA supercoiling, BMS events can be recovered provided that reaction times are long.

It is difficult to quantify the intermolecular reaction using the electrophoresis assay because of the background smear near the top of the gel. However, the donor and target plasmids used for the target titration were identical to those used for the *in vitro* hop assay. Small aliquots of the reaction were therefore retained for transformation. The resulting quantification confirmed that intermolecular integrations increase with target concentration and that at all target concentrations a nicked donor performs more intermolecular integrations than a supercoiled donor (Figure 2B). This difference was low (less than 2-fold), but reproducible. However, since the overall efficiency of integration is lower with the nicked donor, which is evident from the persistence of the ETF, the actual fraction of intermolecular integration events over intramolecular integration events is higher with the nicked donor than what is detected in this assay.

### Target DNA Topology Affects Integration

**Figure 3:**
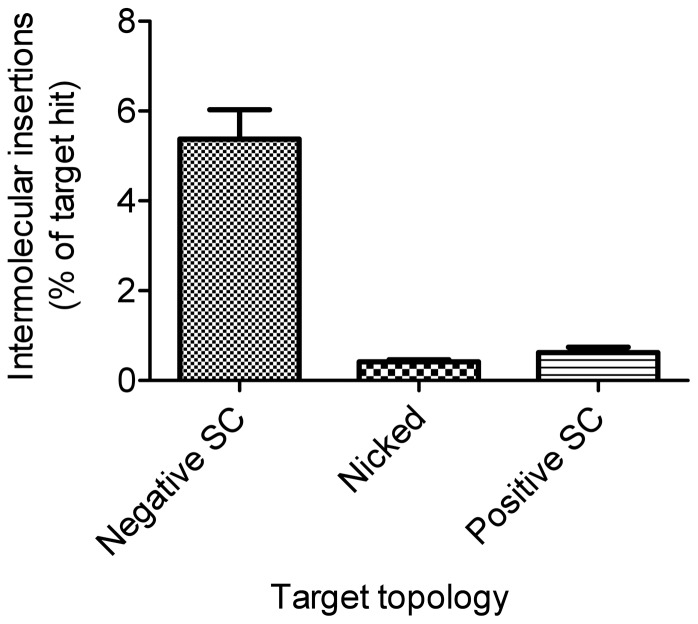
The topology of the target affects integration. Transposition reactions containing 1 nM supercoiled transposon donor (pRC704) were performed in the presence of a 1 nM target plasmid (dimeric pACYC184) that was either negatively supercoiled (SC), nicked, or positively supercoiled. Intermolecular insertions of the transposon into the target plasmid were recovered by genetic transformation in *E. coli*. The percentage of target plasmids integrated by a transposon is plotted.

The target DNA in the experiments described so far was in the supercoiled form. To investigate whether the topology of the target is a significant factor for transposon integration, the *in vitro* hop assay was used to quantify the relative efficiency of negatively supercoiled, nicked and positively supercoiled targets (Figure 3). Intermolecular integration events obtained with a negatively supercoiled target were over 10-fold more numerous than with a nicked and positively supercoiled target, indicating that negatively supercoiled DNA is a much better target than nicked and positively supercoiled DNA. The increased intermolecular events obtained with the negatively supercoiled target are presumably at the expense of the intramolecular events, which are the majority of the products. This helps to explain why an ETF from a nicked donor, which has a low level of supercoiling, if any, is less reactive and performs less intramolecular integrations than that from a supercoiled donor (Figure 2A). It also suggests that the higher levels of intermolecular integrations obtained after 24 h with a nicked substrate results from a failure to perform intramolecular integration owing to the lack of supercoiling in the ETF.

### Target Sequence Bias

**Figure 4:**
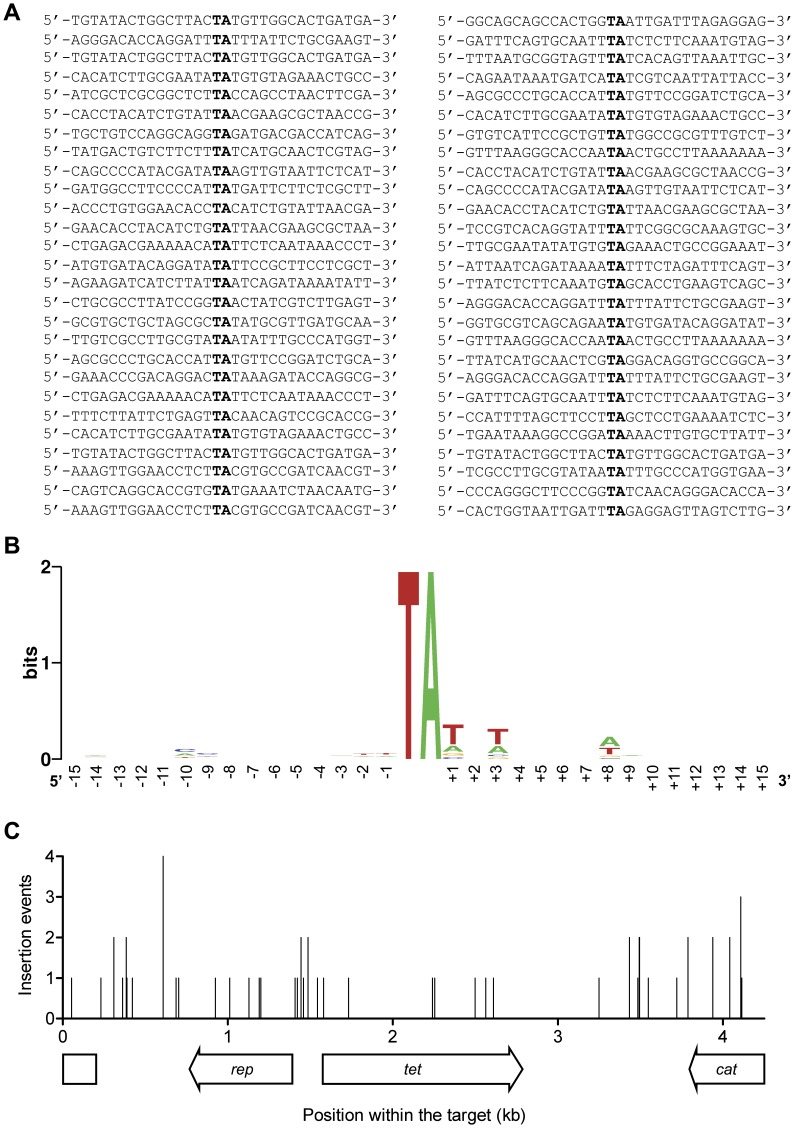
Target site bias. **A**, 54 integration events into a supercoiled pACYC184 target were selected at random from the experiment in Figure 3. The junctions between the transposon and the target were analyzed by DNA sequencing. All 54 insertions were precise and the transposon ends were flanked by duplicated TA dinucleotides. The sequence of the integration sites were centered on the target TA and aligned. **B**, The target sites shown in A were used to generate a logo representing the bias in target site selection in the vicinity of the TA. The logo was created by the program WebLogo [44]. **C**, The position of the 54 integration sites was plotted on a map of the pACYC184 target. *cat*, chloramphenicol resistance marker; *tet*, tetracycline resistance marker; *rep*, origin of replication. Integration events are represented by vertical bars. The target plasmid was a dimeric version of the pACYC184 plasmid, which allowed the detection of integration events into the *cat* gene and the origin of replication.

Quantification in the *in vitro* hop assay is achieved by counting bacterial colonies that result from transformation with intermolecular insertion products. To determine the sequence specificity of *Hsmar1* insertion we selected 54 clones at random from the experiment with the negatively supercoiled target in Figure 3 and sequenced the junctions (Figure 4A). All of the insertions were in a TA dinucleotide. The sequences of the 15 bp on either side of the insertion sites were used to produce a sequence logo (Figure 4B). This revealed that the local DNA sequences immediately surrounding the TA dinucleotide confer very little target specificity. To determine the target specificity over longer distances, the insertion points were plotted on a map of the target plasmid (Figure 4C). Most of the insertions were well distributed throughout the plasmid. However, some sites received more than one insertion. To address whether the observed distribution is statistically different from what would be expected if target site selection was random we compared the data to a Poisson distribution. There are 188 TA dinucleotides within the pACYC184 target. Therefore, each TA has a probability of 0.29 to be present in the set of 54 target sites. According to the Poisson distribution, the probability that any single target site will receive 3 or 4 randomly selected integration events in a set of 54 is 0.56 and 0.04, respectively. Since we recovered one example of each type, it appears that target selection is not entirely random.

### Intramolecular Target Selection

**Figure 5:**
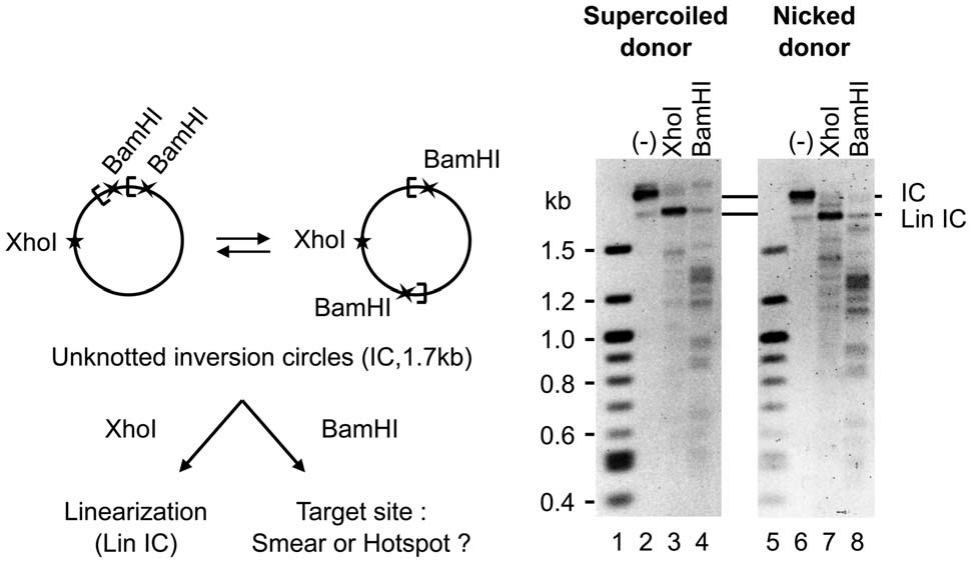
Target site selection during intramolecular integration is not affected by the topology of the transposon. Transposition reactions were performed using a negatively supercoiled or nicked transposon donor pRC650, which encodes a 1.7 kb transposon. The unknotted inversion circle (IC) was gel purified, digested by the restriction enzyme XhoI or BamHI and analyzed by gel electrophoresis. XhoI linearizes the IC (Lin IC). Two BamHI sites are situated close from the transposon ends. Random target site selection will generate a smear while bands into the smear represent hot spots of integration into the transposon. SYBR Green I stained 1.1% agarose gels are shown. The pattern of target site selection was similar with the supercoiled and the nicked donor DNA.

It is conceivable that some of the bias towards intramolecular transposition could arise from the presence of an insertion hot spot in the artificial mini-*Hsmar1* transposons, which all contain a kanamycin resistance marker. To address this issue, the transposon inversion circle product was purified from a gel and analyzed by restriction digestion (Figure 5). The transposon contains a single XhoI recognition site and cleavage with this enzyme linearizes the inversion circle. The transposon also has two BamHI sites located close to the transposon ends. If the intramolecular insertion points are completely random, digestion with BamHI will produce a continuous smear of fragments of different lengths. Any bands within this smear will reveal insertion hot spots. Several bands were evident within the smear (Figure 5). However, these were well distributed, indicating that no major hot spots were present. Finally, the pattern of bands was similar for inversion circles derived from supercoiled or nicked donors, indicating that supercoiling in the target is not a major determinant of site bias.

## Discussion

### Donor and Target Topology Affect Integration

DNA supercoiling accelerates synapsis of the *Hsmar1* transposon ends by increasing the probability of productive collision events between ends that have the appropriate inverted repeat configuration [7]. This is consistent with the experiment in Figure 1C where intermolecular transposition events accumulated more quickly with a supercoiled donor than a nicked donor. However, the effect of the transposon donor topology on the reaction has two features that cannot be explained by the accelerating effects of DNA supercoiling on synapsis. Firstly, if allowed sufficient time to reach completion, a nicked donor yields higher levels of intermolecular integrations than a supercoiled donor (Figures 1C and 2B). Secondly, a transposon excised from a nicked donor suffers from an integration defect (ETF, Figure 2A). Clearly, the integration step is affected by the topology of the transposon donor. However, we also found that negatively supercoiled DNA is the preferred target of the *Hsmar1* transposon (Figure 3). Since the majority of integration events in the *in vitro* reaction are intramolecular, it is likely that the absence of significant levels of supercoiling in excised transposon derived from a nicked donor is at least partially responsible for the integration defect. Indeed, the integration defect of the nicked transposon could be partially restored in the presence of elevated levels of supercoiled target (see ETF with nicked donor, Figure 2A). Furthermore, the target preference for negatively supercoiled DNA suggests that the propensity of the nicked donor to perform intermolecular integration events is the result of the nicked transposon having a lower tendency to integrate into itself than the supercoiled transposon. Indeed, the decrease of intramolecular insertion events with the nicked transposon at high concentrations of negatively supercoiled target was clearly observable (see Intra. with nicked donor, Figure 2A).

### Regulation of Mariner Transposition by DNA Supercoiling

*Hsmar1* transposition is sensitive to DNA topology, both at the level of transposon excision and integration (ref. [7] and this work). We have previously suggested that the stimulatory effects of DNA supercoiling on transposon excision may represent a mechanism to couple transposition to cellular events [7]. This is a well-established property of bacterial transposons, as exemplified by Tn*10*, which is sensitive to supercoiling as well as the global regulators IHF and H-NS [34]. In the case of *Hsmar1*, excision of a transposon located in front of a replication fork would be inhibited by positive supercoiling. This could promote transposon amplification by favoring transposition after the passage of the replication machinery. Transposon excision may also be activated by free negative supercoils formed behind transcription bubbles or by eviction of nucleosomes associated with episodes of chromatin remodeling.

The target preference for negatively supercoiled DNA suggests that transposon integration may also be regulated in response to the dynamics of the nucleus. It is conceivable that the excised transposon preferably interacts with free supercoiled DNA that transiently forms during DNA metabolic processes. However, *Sleeping Beauty* and *Hsmar1* do not appear to have an insertion bias for active genes, suggesting that transcription is not a predominant factor for transposon integration [11], [35]. Moreover, transposons that target transcribing DNA are likely to be more disruptive than transposons that target silent genomic regions. If a transposon were to target negative supercoils formed during DNA replication, it would be expected to integrate behind replication forks. However, in order for a transposon to increase its copy number it would be more advantageous if it were to integrate in front of replication forks. Whether free supercoiled DNA provides *in vivo* targets for *Tc1-mariner* transposons is unknown but it is intriguing to ask whether the preference for supercoiled targets could provide a selective advantage.

An alternative possibility is that the effect of DNA supercoiling on target interactions described here is not related to a mechanism to activate transposon integration in response to chromatin dynamics but instead reflects a fundamentally different aspect of the reaction. Transposon integration is in principle reversible because the strand transfer reaction is isoenergetic. It has been suggested that transposases and integrases ensure the irreversibility of the integration step by introducing a bend in the target [36], [37]. The mechanical strain in the target would be released during the strand transfer reaction and produce a displacement of the newly formed phosphodiester bond away from the active site to prevent the reverse reaction. This was first suggested by the crystal structure of integration intermediates of the foamy virus intasome [38]. A comparison of the pre- and post-catalytic integration complexes showed that the position of the scissile phosphodiester bond is shifted away from the active site after catalysis, making it an unlikely substrate for subsequent nucleophilic attack [36].

The propensity to bend the target site appears to be a common theme in the DDE/D family of integrases and transposases. A similar DNA bend is observed in the recent structure of the Mu target capture complex [39]. Moreover, biochemical analyses have shown that the target site of Tn*10* is bent upon capture by the transpososome [40]. Furthermore, the *Mos1* post-cleavage paired-ends-complex structure suggests that the *mariner* transpososome may also bind the target [41]. Indeed, the structure displays an additional pair of transposon ends that interacts in a non-sequence specific manner with the transpososome and has an angular geometry similar to the foamy virus intasome target. Finally, correlations drawn between preferred targets of *Sleeping Beauty* and predicted physical properties of integration sites suggested that DNA bendability favors target site selection [11]. However, no correlation could be drawn between preferred targets of *Mos1* and the predicted physical properties of these sites [14], [17]. By a similar approach, we could find no correlation between the preferred target sites of *Hsmar1 in vitro* and the predicted curvature and bendability of the target, although this analysis was probably hampered by the lack of statistical power (not shown).

Another interesting question that remains to be investigated with *Tc1-mariner* elements is how nucleosomes influence target site selection. Nucleosomes have different impacts on the integration bias of viruses and transposons. For example, the human immunodeficiency virus and murine leukemia virus integrate preferentially nucleosome-occupied DNA *in vitro*, presumably due to their propensity to target bendable DNA or sites that are bent by protein complexes [42]. The *Ty1* retrotransposon also preferentially integrates into nucleosome-occupied targets whereas the *hAT* transposon *Hermes* and the *Tf1* retrotransposon integrate preferentially into nucleosome-free regions [21], [22], [23].

Supercoiling can affect DNA transactions by several mechanisms. Supercoiling increases the local concentration between any two sites. The right-handed intertwining of plectonemic negative supercoils can juxtapose two sites in a specific angular configuration. Negative supercoiling also underwinds the DNA and increases its bendability. We have previously shown that the first two factors – concentration and orientation – accelerate synapsis of the *Hsmar1* transposon ends [7]. In contrast, the stimulatory effect of DNA supercoiling on transposon integration is likely to be due to the underwinding or the bendability of DNA. However, our observations do not allow us to clearly discriminate between these two factors. Because positive supercoiling does not promote target interactions, we would expect the underwinding of the DNA to be a predominant factor. In this case we would expect nucleosomal DNA to provide poor targets for *mariner* insertions because DNA is probably overwound at the surface of nucleosomes [43].

### Target Sequence Bias

The nucleotide sequence at the vicinity of the target TA usually provides little specificity for *Tc1-mariner* transposons [11], [12], [13], [14]. Nevertheless, consensus integration sites have been reported for *Mos1* (‘TATA’ or ‘TAxTA’) and *Sleeping Beauty* (palindromic AT repeat ‘ATATATAT’) [11], [14], [17]. *Hsmar1* also appears to target preferentially TA dinucleotides that are flanked by A-T base pairs (Figure 4B). However, as in the case of *Himar1*, this preference is mild and no consensus integration site can be derived [13]. Intriguingly, the alignment of *Hsmar1* target sites revealed some sequence bias for the nucleotides located about one helix turn away from the TA target, possibly reflecting interactions between the transpososome and these nucleotides. Although the distribution of *Hsmar1* integration events was not random, no major integration hotspots were detected (Figures 4C and 5). *Mos1* appears to display a stronger integration bias than *Hsmar1*. The chloramphenicol acetyltransferase (*cat*) gene from Tn*9* was reported to be a strong hotspot for *Mos1* insertions [17]. In contrast, *Hsmar1* does not display an integration bias for this gene and integration events within a plasmid that contained the *cat* gene were well distributed throughout the plasmid (Figure 4C). The *cat* gene requires DNA supercoiling to provide an integration hot spot for *Mos1* [17]. In contrast, the stimulatory effect of DNA supercoiling on *Hsmar1* target interactions appears to be general. The pattern of intramolecular integration sites also suggested that the target selection bias is unaffected by the topology of the target (Figure 5).
